# The Action of the Voluntary Muscles

**Published:** 1862-06

**Authors:** Louis Mackall


ART VI.—*The Action of the Voluntary Muscles; by* LOUIS MACKALL, M. D. (*Extract from an unpublished work.*)

[CONTINUED.]

79. If what we have suggested be admitted to be the true law of muscular action—if innervation, or the determination of the nerve-fluid to a muscle, is attended with the active elongation of its fibres, there is no difficulty whatever in comprehending or in offering a rational explanation of the phenomenon before us. The animal, it will be understood, determines its nerve-fluid to the lingual muscles, and the tongue is extended to the distance mentioned, simply by the active elongation of these muscles. The food is also drawn into the mouth by the retraction of the same muscles, caused by the withdrawal from them of this fluid.

But what explanation of this phenomenon can be given without the aid of this law? We will give the only one we have met with worthy of any notice, that of Mr. John Hunter, in his own words, taken from the "Illustrated Catalogue of the Hunterian Museum."

"This length of tongue, its extension and contraction, are very singular, and, if well understood, most probably very curious.

The cause and mode of the contraction of its length are not uncommon. The elongation of the tongue in this animal is perhaps like nothing that we are acquainted with in an animal body.

The apparatus for this purpose is a small, rounded body, which passes from the apex of the os linguæ (glosso-hyal) to the bulbous part, and then through the centre of the bulb. The part between bone and bulb consists of two different substances, one a whitish substance, which is the firmest, and appears to be capable of keeping its form; the other is softer and more transparent. That part which passes through the bulb consists only of one substance, and appears to be a sheath for the reception of the os linguæ." The reader will please recollect that the apparatus, here described by Mr. Hunter, is nothing more than the bands and meshes of *cellular tissue*, of which we have spoken above. But let him proceed:

"The first of these (i. e. the whitish, firmer substance) appears to be composed of rings, or something similar, placed obliquely in contrary directions, so as to appear to be two spirals crossing one another. Whether the other or softer substance" [the cellular meshes. L. M.] "has any direction of fibres, I could not observe, but I suspect it is muscular. If I am right in my conjecture of this structure, and of its disposition, it will be no difficult thing to show how it may be elongated; for if these rings are placed transverse, they may be brought so near to one another as to shorten the whole very considerably; and if they allow of being placed almost longitudinally, they must of course lengthen it very considerably, and this position can easily be produced by muscles, which I take the pulpy substance to be.

The contraction of the tongue is owing to a degree of elasticity; but this appears to be only in the cellular membrane, acting as an assistant to the muscular. The muscular contraction is owing to two muscles, one on each side of the tongue; each arises from the os hyoides on the inside of the os linguale, and passes along the side of the tongue to its bulbous part; but before it gets to the bulbous part it spreads itself all round.

In the centre of each of these two muscles passes a considerable nerve to the bulbous part, and also two arteries. When the two muscles act, they draw the tongue back upon the os linguale, which, as it were, passes through the middle elongator, then through the centre of the bulb, till the whole tongue is retracted. Although this middle body is drawn upon the os linguæ, yet it does not appear to be a hollow like a pipe; it rather appears to be filled with a very ductile cellular membrane, as in every part of the elongating division of the tongue, in order to allow of the great difference in the situation of parts with respect to one another." (Hunterian manuscript.)

80. The ant-eater (Myrmeco-phaga) furnishes a remarkable instance of the action of the muscles of this class. The tongue of this animal is composed of two distinct muscles, having different origins, and a different disposition of fibres. To simplify the subject, we may say one of these muscles arises from the sternum, and passing along through the muscles on the anterior surface of the neck, terminates at the tip of the tongue—its fibres being all arranged longitudinally; the other muscle arises from the os hyoides, passes around the former longitudinal muscle in close spirals, forming a sort of case or sheath for it, and terminates with it, at the end of the tongue.

When quiescent, or in a state of inaction, the tongue of the ant-eater is probably about six or eight inches long; but when thrust into the ant-hills to secure its prey, Naturalists tell us, it is elongated or protruded to the extent of *seventeen* or *eighteen* inches.*

The object attained by this curious arrangement of the muscles, in accordance with our view of muscular action, is evidently the free and unimpeded extension of the longitudinal muscle to its full capacity, by preserving its fibres, nerves, and blood-vessels from external pressure, while threading the narrow passages of the ant-hill through which it is forced. This object is fully accomplished by the action of the spiral muscle, which tends to dilate these passages, to enlarge and keep open the space enclosed by it, in which the longitudinal muscle acts.

In the absence of this view, Physiologists have been forced to adopt the very absurd supposition, that the tongue of the ant-eater is protruded by the *contraction* of its *spiral muscle!*

81. The woodpecker, as is well known, feeds on the grubs that burrow into the limbs and bodies of trees. The burrows are first exposed by pecking through the bark with its strong, sharp bill, and then the long tongue, like a flexible probe, is thrust in, as the ant-eater's into the ant-hill, and the grub being harpooned, as it were, is dragged forth.

---

* Curvier's Animal Kingdom, edited by Griffith, vol. iii, p. 300.

To adapt the organ to the purposes mentioned, the anterior portion of the tongue—I refer more particularly to that of the species called the Flicker, (Picus aureatus)—is composed of a horny, spear-shaped, barbed point; from this point there arise two muscles, tendinous at each extremity, but with fleshy bellies or middles—their fibres longitudinal; these muscles pass around the base of the cranium, one on each side, and then rest with free extremities in a groove or closed passage formed between the skin and cranium, and extending over the middle of the head from behind forward until it reaches the base of the upper mandible. Besides these, two other muscles arise, one from the ramus of the lower mandible on each side, and pass around the longitudinal muscles, forming a sheath for them through their whole length. The arrangement of these muscles are strikingly similar to that of the muscles of the tongue of the ant-eater, and doubtless their functions are the same. When the longitudinal muscles are elongated, the upper bill, which is then applied to the tree, becomes the basis or point of resistance, from which the tongue is projected into the burrows; the other or spiral muscles acting, as suggested, when speaking of that of the ant-eater, to preserve the freedom of the motion of the former, by protecting from external pressure the blood-vessels and nerves with which they are largely supplied.

The notion suggested by some, that the tongue is jerked forward by the contraction of the last-mentioned muscles, is absurd; as the means suggested are clearly inadequate to the end proposed. The contraction of any portion of these muscles could not effect a motion of the tongue to the extent of one inch, but, if contracted, the portions anterior to their origins would draw the tongue back, and counteract the effect of the contraction of the portions of these muscles that are posterior to their origins, so that no effect of the kind suggested, could result from the contraction of these muscles. The tongue is thrust into the burrows, probably to a distance of from five to eight inches, judging from the extensibility of the organ in the living or recently killed bird.

The longitudinal muscles of the tongue are commonly regarded as the cornua of the os hyoides, but I think it more rational to regard them as muscles (as their appearance clearly indicates) belonging to this class, attached at one extremity with the rest of the organ free, to be extended or retracted at will.

82. The "Aij-Aij" is an animal whose habitat is confined to Madagascar. It presents several points in its natural history closely resembling those of the woodpecker. Like this, it feeds on a grub that burrows in the bodies of trees in that island; and it exposes these burrows by tearing off the bark with its teeth. The animal then introduces a long, flexible probe, and pulls out the grub. This probe, however, is not the tongue; but one of the fingers of one of the fore-paws, which is curiously adapted in its structure to this purpose. The anatomy of this organ is not generally known, but I presume, from the office it is said to perform, that it is similar to that of the tongues of the ant-eater and of the woodpecker that we have just described. In its retracted state it may present the appearance of a "shrunken" or "etrophied" member, as is represented; but its muscles are, I doubt not, well developed, with large blood-vessels and nerves, and capable of an extensive action of elongation.

83. The tentacles of some of the lower orders of animals are so similar in structure, in their action, and in the purposes they subserve, to the tongues of the higher orders, that we are induced to regard them as their analogues. These organs are composed of muscles with longitudinal fibres, that are attached by one extremity around the outer margin of the mouth, and are extended or actively elongated for the purpose of collecting and of bringing into the mouth, the food or prey that may be floating in the element these animals inhabit. When the animals are reposing, or not feeding, these organs are retracted; but when engaged in taking their food, the tentacles are innervated, and actively elongated or extended. In some instances, as in the Physalus, the Enychoteuthis, &c., this extension is carried to a distance of six feet or more.* For a more minute account of the structure and office of these organs, I refer to the works on comparative Anatomy and Physiology. Our theory of muscular action offers to the intelligent student a number of suggestions that will be found of great use to him in his efforts to understand the more complicated structure and action of some of these organs.

---

Large Doses of Quinia in Typhoid Fever.—Dr. C. P. Herrington, of Ashland, Pa., writes us as follows, relative to his method of treating this fever by the administration of large doses of quinia: "The dose is from four to eight grains *every hour* during the remissions, which are always very *distinct* in the morning. We never have typhoid fever in our part of the country without *remissions* occurring every morning."—*Medical and Surgical Reporter.*

* General Structure of the Animal Kingdom, by T. Rymer Jones.

